# Supplementary material for: Rapid protection induced by a single-shot Lassa vaccine in male cynomolgus monkeys
Source: Nat Commun. 2023 Mar 11;14:1352. doi: 10.1038/s41467-023-37050-6 (PMC10008018; doi:10.1038/s41467-023-37050-6)
Supplement: Supplementary file 1 — Supplementary Information [file 41467_2023_37050_MOESM1_ESM.pdf]

Supplementary Information

Rapid protection induced by a single-shot Lassa vaccine in cynomolgus monkeys

Mathieu Mateo[1,2], Stéphanie Reynard[1,2], Natalia Pietrosemoli[3], Emeline Perthame[3], Alexandra Journeaux[1,2], Kodie Noy[1,2], Clara Germain[1,2], Xavier Carnec[1,2], Caroline Picard[1,2], Virginie Borges-Cardoso[1,2], Jimmy Hortion[1,2], Hélène Lopez-Maestre[3], Pierrick Regnard[4], Lyne Fellmann[4], Audrey Vallve[5], Stéphane Barron[5], Ophélie Jourjon[5], Orianne Lacroix[5], Aurélie Duthey[5], Manon Dirheimer[6], Maïlys Daniau[7], Catherine Legras-Lachuer[7], Caroline Carbonnelle[5], Hervé Raoul[5], Frédéric Tangy[8], and Sylvain Baize[1,2*]

[1]Unité de Biologie des Infections Virales Emergentes, Institut Pasteur, 69007, Lyon, France

[2]Centre International de Recherche en Infectiologie (CIRI), Université de Lyon, INSERM U1111, Ecole Normale Supérieure de Lyon, Université Lyon 1, CNRS UMR5308, 69007, Lyon, France

[3]Institut Pasteur, Université Paris Cité, Bioinformatics and Biostatistics Hub, Paris, France

[4]SILABE, Université de Strasbourg, fort Foch, Niederhausbergen, France

[5]Laboratoire P4 INSERM – Jean Mérieux, INSERM US003, 69007, Lyon, France

[6]INSERM, Délégation Régionale Auvergne Rhône-Alpes, 69500, Bron, France

[7]Viroscan 3D SAS, Trévoux, France

[8]Vaccine Innovation Laboratory, Institut Pasteur, 75015, Paris, France

*Corresponding author: sylvain.baize@pasteur.fr

Temperature			Score
	Day T°C	ΔT°	0 pt: ΔT°≤±1
			1 pt: 1<ΔT°≤1.7
			2 pts: 1.7<ΔT°≤2.2
			3 pts: ΔT°>±2.2
**Weight loss**			**Score**
	Day weight (kg)	Δ weight	0 pt: ΔW≤5.4%
			1 pt: 5.4%<ΔW≤7.5%
			2 pts: 7.5%<ΔW≤10%
			5 pts: Δ10>10%
**Bleeding**			**Score**
			0 pt: nothing remarkable (NR)
			3 pts: bleeding
**Petechiae**			**Score**
	Petechiae % of body		0 pt: NR
			1 pt: 1% to 19%
			2 pts: 20% to 49%
			3 pts: > 49%
**Stool**			**Score**
			0 pt: NR
			1 pt: loose stool
			2 pts : diarrhea
			5 pts: mucous and fibrinous diarrhea
**Responsiveness**			**Score**
			0 pt: normal activity
			2 pts: general loss in tone, sightly slow in motion
			4 pts: more often seated than perched, still interacts with its environment
			6 pts: weak, stays seated and seeks isolation. Decreased interactions with its environment. Difficulty moving and reflexes reduced.
			10 pts: prostration and no more interaction with the environment.
**Dehydration**			**Score**
			0 pt: NR
			1 pt: dehydration
**TOTAL SCORE**			**Euthanasia if total score ≥15***

Table S1. Scoring and endpoints for animal studies. Euthanasia was also performed when an animal reached one of the following endpoints: strong prostration, body temperature below 35.8 °C, no awakening at 150 minutes post anesthesia, or if the animal was moribund.

Supplementary Figures

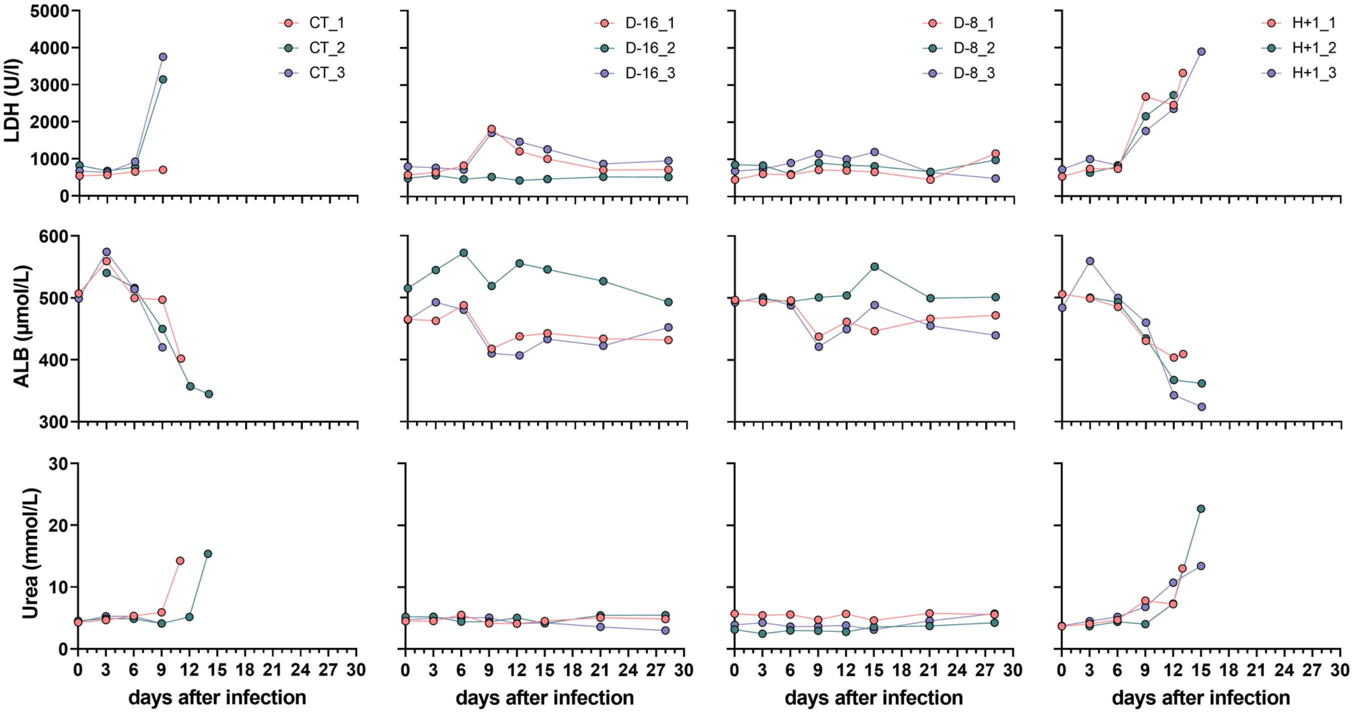

Supplementary Figure 1. Biochemical analyses of plasma samples from cynomolgus monkeys after LASV challenge. Analysis of lactate dehydrogenase (LDH), albumin (ALB), and urea plasma concentrations during the course of LASV infection. Individual data are presented for each monkey. Source data are provided as a Source Data file.

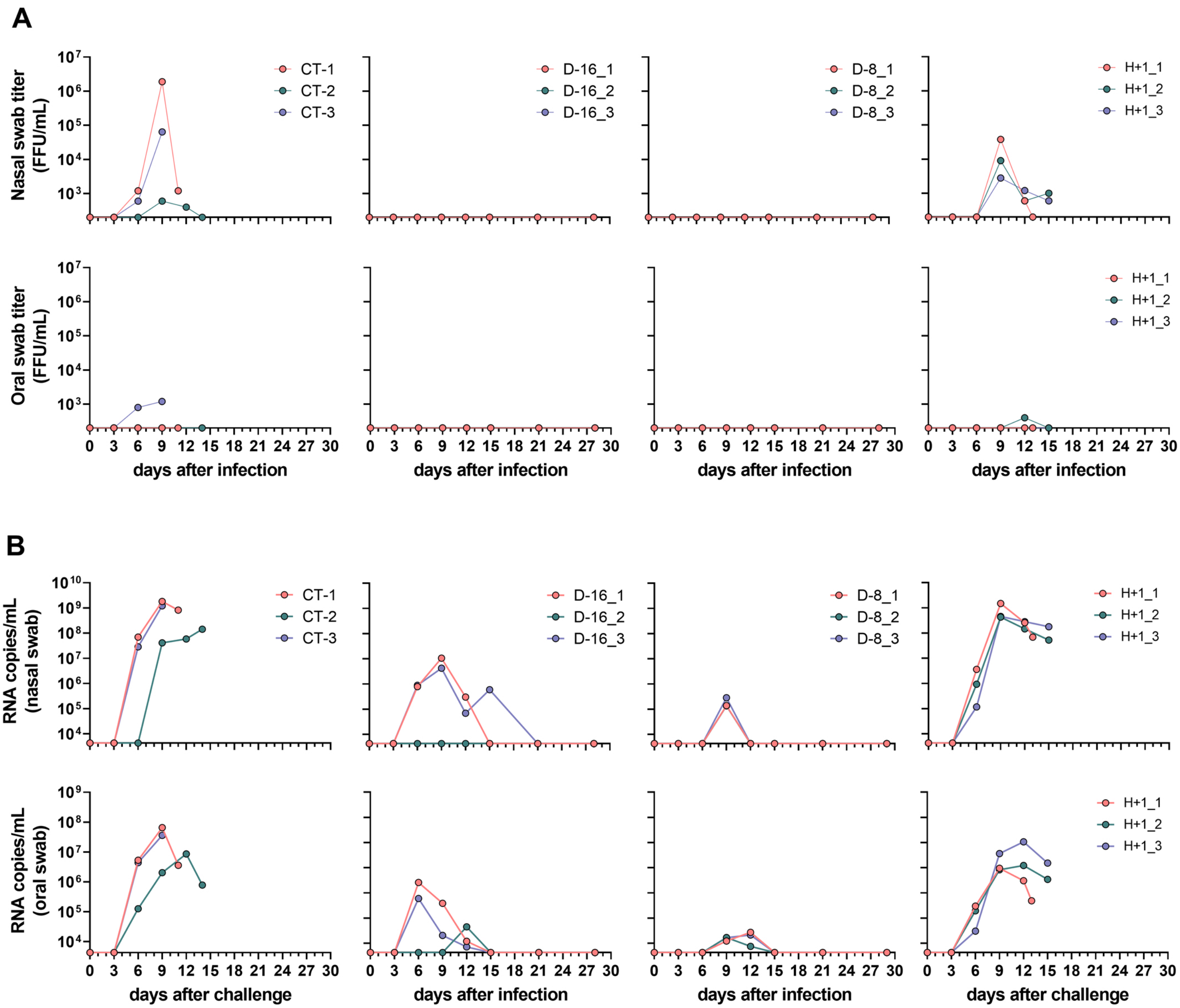

Supplementary Figure 2. LASV replication in nasal and oral swabs of cynomolgus monkeys after LASV challenge. Quantification of (**A**) infectious LASV titers (in FFU per milliliter) and (**B**) LASV viral loads (in RNA copies/mL), measured in nasal and oral swabs collected over the course of LASV infection in unvaccinated control animals (CT), animals vaccinated 16 days (D-16) or 8 days (D-8) before the LASV challenge, or animals vaccinated one hour after the LASV challenge (H+1). Individual data are presented for each animal. Source data are provided as a Source Data file.

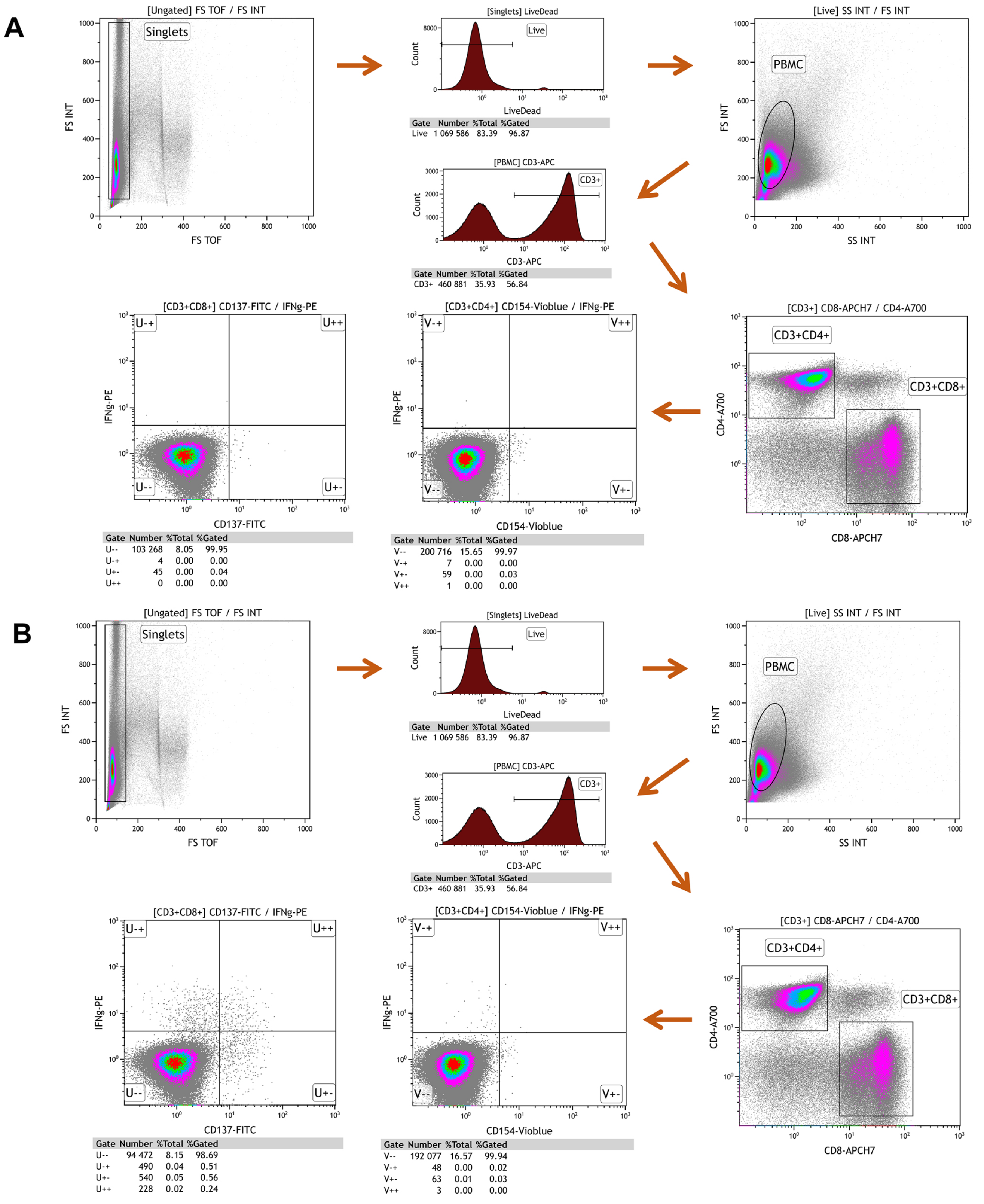

Supplementary Figure 3. Gating strategy for intracellular cytokine staining analysis. The gates used to quantify IFNγ-producing and CD154- or CD137-expressing T cells are presented for an unstimulated sample from an immunized animal (**a**) and for the same sample stimulated with LASV GPC peptides (**b**). FSCint/FSCtof was used to select singlets (*singlets* gate). Then, dead cells are excluded using live-dead staining (*live* gate). Lymphocytes were selected using FSCint/SSCint parameters (*PBMC* gate). CD3+ T cells were selected among PBMCs (*CD3+* gate). Then, CD4+ and CD8+ T cells were selected using CD4/CD8 staining (*CD4+* and *CD8+* gates). Finally, the percentage of IFNγ-producing and CD154-expressing CD4+ and CD137-expressing CD8+ T cells is determined using a quadrant in the IFNγ/CD154 and IFNγ/CD137 dot plots.

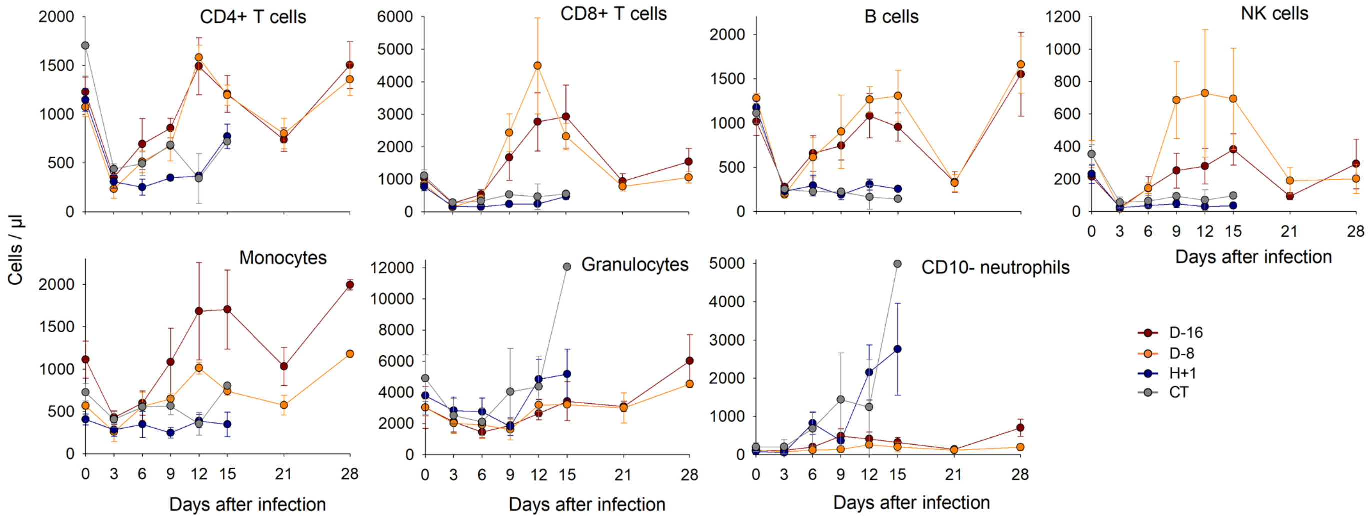

Supplementary Figure 4. Blood counts after LASV challenge. Numbers of $CD4^{+}$ and $CD8^{+}$ T cells, NK cells, B cells, monocytes, granulocytes, and CD10- neutrophils per microliter of whole blood according to the time after the LASV infection measured by flow cytometry. Data are presented as mean values +/- SEM of n=3 independent samples. Source data are provided as a Source Data file.

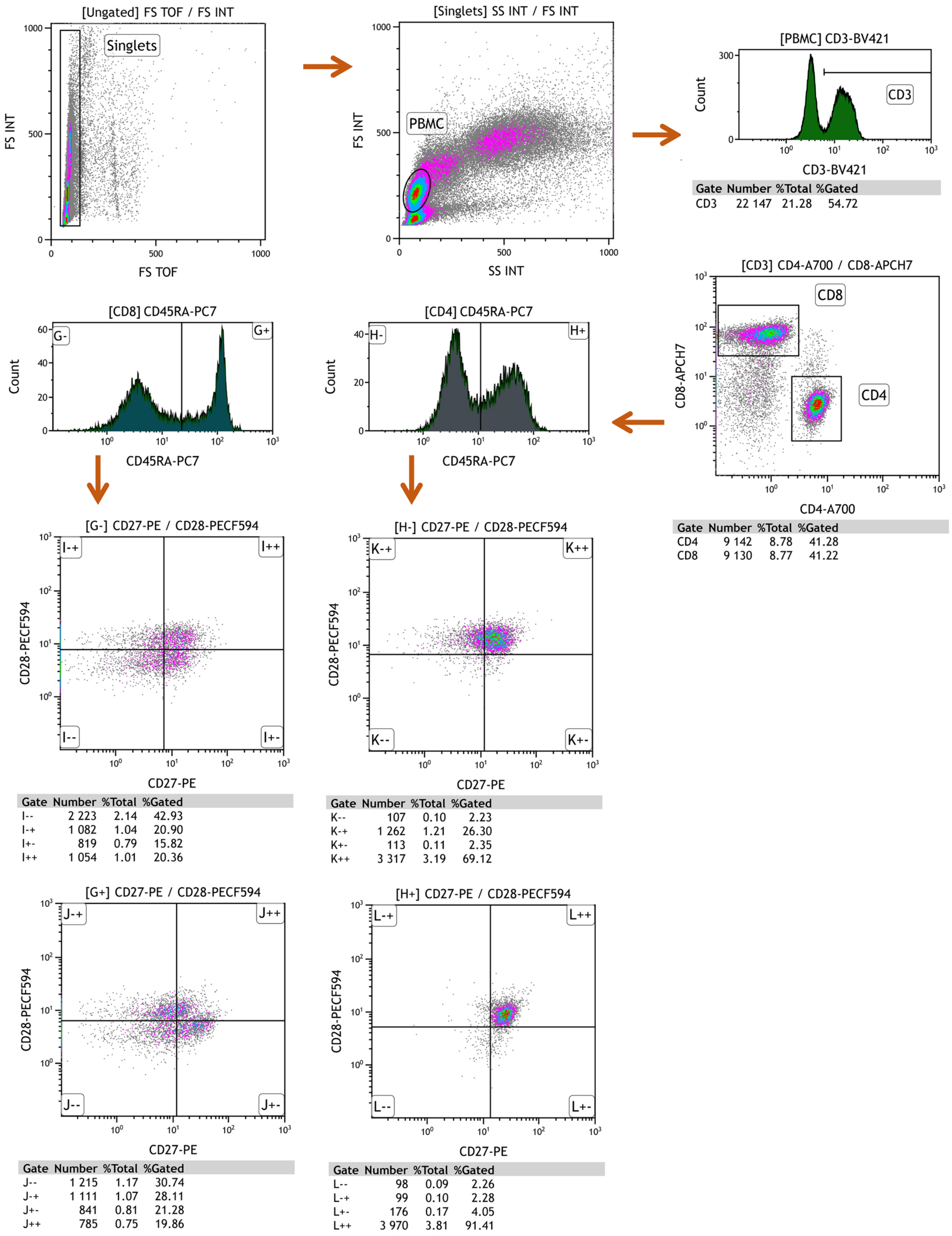

Supplementary Figure 5. Gating strategy for T cell phenotype analyses. The gates used to quantify the different populations of CD4+ and CD8+ according to the memory phenotype are presented. FSCint/FSCtof was used to select singlets (*singlets* gate). PBMCs were selected using FSCint/SSCint parameters (*PBMC* gate). CD3+ T cells were selected among PBMCs (*CD3+* gate). Then, CD4+ and CD8+ T cells were selected using CD4/CD8 staining (*CD4+* and *CD8+* gates). Finally, the percentage of CD45RA, CD27, and CD28 expression by CD4+ and CD8+ T cells is presented. The same strategy was used for analysis of CD69, CD134, CD279, NKp80, KI67, GrzB, and perforin expression.

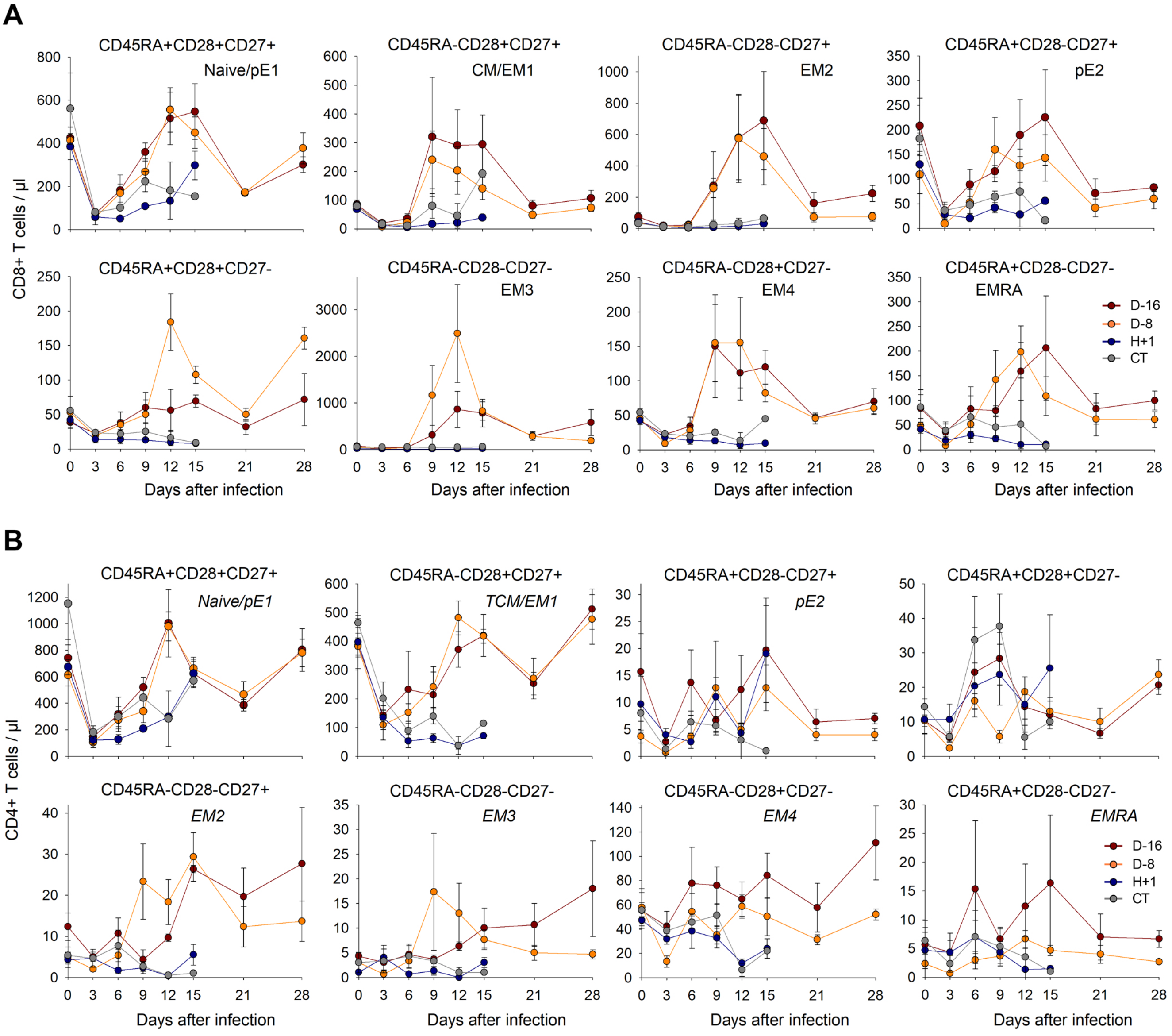

Supplementary Figure 6. Evolution of CD8$^+$ and CD4$^+$ T-cell subtypes during the course of LASV infection. (**A**) Number of cells for CD8$^+$ T-cell memory subtypes per microliter of whole blood according to the time after LASV infection measured by flow cytometry. Data are presented as mean values +/- SEM of n=3 independent samples. (**B**) Number of cells for CD4$^+$ T-cell memory subtypes per microliter of whole blood according to the time after LASV infection measured by flow cytometry. Data are presented as mean values +/- SEM of n=3 independent samples. Source data are provided as a Source Data file.

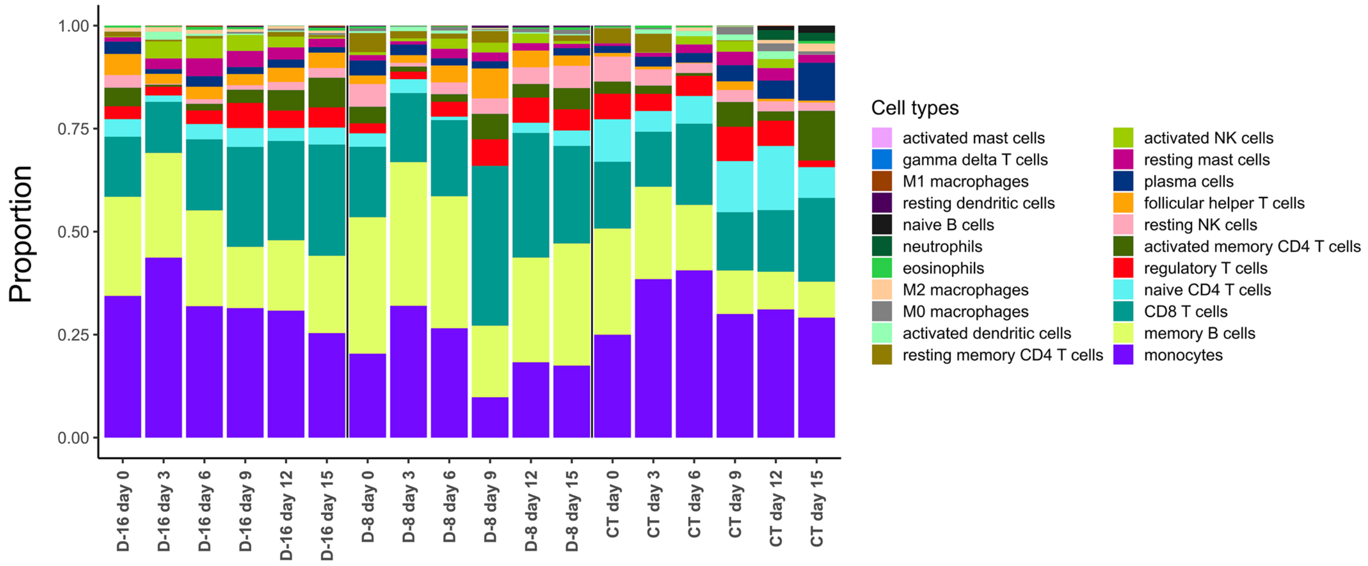

Supplementary Figure 7. Evolution of the cell-type composition estimates in PBMC samples over time based on transcriptome data. The inferred proportion of 22 cell types in PBMC samples collected at various time points after the LASV infection.
