# Supplementary material for: Rapid protection induced by a single-shot Lassa vaccine in male cynomolgus monkeys
Source: Nat Commun. 2023 Mar 11;14:1352. doi: 10.1038/s41467-023-37050-6 (PMC10008018; doi:10.1038/s41467-023-37050-6)
Supplement: Supplementary file 3 — Reporting Summary [file 41467_2023_37050_MOESM3_ESM.pdf]

nature portfolio

Corresponding author(s): Sylvain BAIZE

Last updated by author(s): 18/01/2023

Reporting Summary

Nature Portfolio wishes to improve the reproducibility of the work that we publish. This form provides structure for consistency and transparency in reporting. For further information on Nature Portfolio policies, see our Editorial Policies and the Editorial Policy Checklist.

Statistics

For all statistical analyses, confirm that the following items are present in the figure legend, table legend, main text, or Methods section.

n/a	Confirmed	
☐	☒	The exact sample size (*n*) for each experimental group/condition, given as a discrete number and unit of measurement
☐	☒	A statement on whether measurements were taken from distinct samples or whether the same sample was measured repeatedly
☐	☒	The statistical test(s) used AND whether they are one- or two-sided<br>*Only common tests should be described solely by name; describe more complex techniques in the Methods section.*
☐	☒	A description of all covariates tested
☐	☒	A description of any assumptions or corrections, such as tests of normality and adjustment for multiple comparisons
☐	☒	A full description of the statistical parameters including central tendency (e.g. means) or other basic estimates (e.g. regression coefficient) AND variation (e.g. standard deviation) or associated estimates of uncertainty (e.g. confidence intervals)
☐	☒	For null hypothesis testing, the test statistic (e.g. F, t, r) with confidence intervals, effect sizes, degrees of freedom and P value noted<br>*Give P values as exact values whenever suitable.*
☒	☐	For Bayesian analysis, information on the choice of priors and Markov chain Monte Carlo settings
☒	☐	For hierarchical and complex designs, identification of the appropriate level for tests and full reporting of outcomes
☒	☐	Estimates of effect sizes (e.g. Cohen's *d*, Pearson's *r*), indicating how they were calculated

Our web collection on statistics for biologists contains articles on many of the points above.

Software and code

Policy information about availability of computer code

Data collection	Biochemical analyses were performed on plasma from heparin lithium blood tubes using a Pentra C200 analyzer (Horiba). RT-qPCR were were performed with a LightCycler480 (Roche). Sequencing of RNA from PBMCs was performed on a NextSeq 500 Flow Cell High OutputSR75 instrument (Illumina). Flow cytometry data were collected using a Gallios cytometer (Beckman Coulter).
Data analysis	Graphs and statistical analyses were performed using GraphPad Prism 9.4.1 or SigmaPlot 14.5 softwares except for transcriptomic data that were analyzed and represented using R software, version 4.0.4. RT-qPCR were analyzed with the LC480 software, 1.5.0 SP4 version (Roche). Flow cytometry analyzes were performed using Kaluza Analysis 2.1 software (Beckman Coulter). The evolution of the transcriptomic signatures in the PBMC samples after LASV challenge was studied using heatmaps for the user-defined list of genes generated with the pheatmap R package v 1.0.12. The boxplots for the expression of the genes of each gene list were produced using the ggplot2 R package v 3.3.6. Mean expression values for each time point were statistically compared to assess their difference using the non-parametric Wilcoxon test and Benjamini-Hochberg correction for multiple testing, as implemented in the ggpubr R package v 0.4.0.

For manuscripts utilizing custom algorithms or software that are central to the research but not yet described in published literature, software must be made available to editors and reviewers. We strongly encourage code deposition in a community repository (e.g. GitHub). See the Nature Portfolio guidelines for submitting code & software for further information.

Data

Policy information about availability of data

All manuscripts must include a data availability statement. This statement should provide the following information, where applicable:

- Accession codes, unique identifiers, or web links for publicly available datasets
- A description of any restrictions on data availability
- For clinical datasets or third party data, please ensure that the statement adheres to our policy

The RNA-seq data generated in this study have been deposited in the Zenodo database under accession code 7547502 [https://doi.org/10.5281/zenodo.7547502]. The primary data generated in this study are provided in the Source Data file.

Human research participants

Policy information about studies involving human research participants and Sex and Gender in Research.

Reporting on sex and gender	N/A
Population characteristics	N/A
Recruitment	N/A
Ethics oversight	N/A

Note that full information on the approval of the study protocol must also be provided in the manuscript.

Field-specific reporting

Please select the one below that is the best fit for your research. If you are not sure, read the appropriate sections before making your selection.

☒ Life sciences ☐ Behavioural & social sciences ☐ Ecological, evolutionary & environmental sciences

For a reference copy of the document with all sections, see nature.com/documents/nr-reporting-summary-flat.pdf

Life sciences study design

All studies must disclose on these points even when the disclosure is negative.

Sample size	Sample size was adapted to be at least n=3 and to respect the maximum of twelve animals for an experiment in the BSL4 facility. With the aim to respect the 3R rule in animal studies (Reduce, Refine, Replace) we stated that a minimum of n=3 should be sufficient to provide confident results on vaccine efficacy in comparison with control animals non-vaccinated and infected with a virus that induce accute haemorragic fever.
Data exclusions	None
Replication	Control animals challenged with Lassa virus behaved like control animals used in previous studies (Mateo, Science Transl. Med., 2019 & 2021), ensuring reproducibility. In three independendant experiments, control animals behaved like in this study. In summary, replication attempts were successful for a total of 12 animals.
Randomization	Animals were allocated to their groups because of their social interactions with the others and there was no significant differences in their age or weight.
Blinding	Blinding was not possible in these experiments because of biosafety constraints in the BSL4 animal facilities. Indeed, as the animals were accomodated by 3 in the same cage, it was not possible to mix animals receiving different treatments to avoid cross-contamination.

Behavioural & social sciences study design

All studies must disclose on these points even when the disclosure is negative.

Study description	*Briefly describe the study type including whether data are quantitative, qualitative, or mixed-methods (e.g. qualitative cross-sectional, quantitative experimental, mixed-methods case study).*
Research sample	*State the research sample (e.g. Harvard university undergraduates, villagers in rural India) and provide relevant demographic*

Research sample	*information (e.g. age, sex) and indicate whether the sample is representative. Provide a rationale for the study sample chosen. For studies involving existing datasets, please describe the dataset and source.*
Sampling strategy	*Describe the sampling procedure (e.g. random, snowball, stratified, convenience). Describe the statistical methods that were used to predetermine sample size OR if no sample-size calculation was performed, describe how sample sizes were chosen and provide a rationale for why these sample sizes are sufficient. For qualitative data, please indicate whether data saturation was considered, and what criteria were used to decide that no further sampling was needed.*
Data collection	*Provide details about the data collection procedure, including the instruments or devices used to record the data (e.g. pen and paper, computer, eye tracker, video or audio equipment) whether anyone was present besides the participant(s) and the researcher, and whether the researcher was blind to experimental condition and/or the study hypothesis during data collection.*
Timing	*Indicate the start and stop dates of data collection. If there is a gap between collection periods, state the dates for each sample cohort.*
Data exclusions	*If no data were excluded from the analyses, state so OR if data were excluded, provide the exact number of exclusions and the rationale behind them, indicating whether exclusion criteria were pre-established.*
Non-participation	*State how many participants dropped out/declined participation and the reason(s) given OR provide response rate OR state that no participants dropped out/declined participation.*
Randomization	*If participants were not allocated into experimental groups, state so OR describe how participants were allocated to groups, and if allocation was not random, describe how covariates were controlled.*

Ecological, evolutionary & environmental sciences study design

All studies must disclose on these points even when the disclosure is negative.

Study description	*Briefly describe the study. For quantitative data include treatment factors and interactions, design structure (e.g. factorial, nested, hierarchical), nature and number of experimental units and replicates.*
Research sample	*Describe the research sample (e.g. a group of tagged Passer domesticus, all Stenocereus thurberi within Organ Pipe Cactus National Monument), and provide a rationale for the sample choice. When relevant, describe the organism taxa, source, sex, age range and any manipulations. State what population the sample is meant to represent when applicable. For studies involving existing datasets, describe the data and its source.*
Sampling strategy	*Note the sampling procedure. Describe the statistical methods that were used to predetermine sample size OR if no sample-size calculation was performed, describe how sample sizes were chosen and provide a rationale for why these sample sizes are sufficient.*
Data collection	*Describe the data collection procedure, including who recorded the data and how.*
Timing and spatial scale	*Indicate the start and stop dates of data collection, noting the frequency and periodicity of sampling and providing a rationale for these choices. If there is a gap between collection periods, state the dates for each sample cohort. Specify the spatial scale from which the data are taken*
Data exclusions	*If no data were excluded from the analyses, state so OR if data were excluded, describe the exclusions and the rationale behind them, indicating whether exclusion criteria were pre-established.*
Reproducibility	*Describe the measures taken to verify the reproducibility of experimental findings. For each experiment, note whether any attempts to repeat the experiment failed OR state that all attempts to repeat the experiment were successful.*
Randomization	*Describe how samples/organisms/participants were allocated into groups. If allocation was not random, describe how covariates were controlled. If this is not relevant to your study, explain why.*
Blinding	*Describe the extent of blinding used during data acquisition and analysis. If blinding was not possible, describe why OR explain why blinding was not relevant to your study.*

Did the study involve field work? ☐ Yes ☒ No

Reporting for specific materials, systems and methods

We require information from authors about some types of materials, experimental systems and methods used in many studies. Here, indicate whether each material, system or method listed is relevant to your study. If you are not sure if a list item applies to your research, read the appropriate section before selecting a response.

Materials & experimental systems

n/a	Involved in the study
☐	☒ Antibodies
☐	☒ Eukaryotic cell lines
☒	☐ Palaeontology and archaeology
☐	☒ Animals and other organisms
☒	☐ Clinical data
☒	☐ Dual use research of concern

Methods

n/a	Involved in the study
☒	☐ ChIP-seq
☐	☒ Flow cytometry
☒	☐ MRI-based neuroimaging

Antibodies

Antibodies used

CD8 and CD4 T-cell, B-cell, NK-cell , monocyte, and granulocyte counts were all determined by flow cytometry using antibodies directed against the following proteins: CD56 (560360, V540 mouse anti-human, clone B159, 1.25 µL), CD3 (560770, V500 mouse anti-human, clone SP34-2, 1.25 µL), CD45 (557803, FITC mouse anti-NHP, clone D058-1283, 5 µL), CD10 (557143, PE mouse anti-human, clone HI10α, 5 µL), CD20 (560735, PE-CyTM7 mouse anti-human, clone 2H7, 1.25 µL), CD4 (560836, Alexa FluorR 700 mouse anti-human, clone L200, 1.25 µL), and CD8 (560179, APC-H7 mouse anti-human, clone SK1, 1.25 µL), all from BD biosciences, and an antibody from Miltenyi Biotec directed against NKp80 (130-094-845, APC mouse anti-human, clone 4A4.D10, 2.5 µL).
For T cell activation assays, samples were then treated with PBS-EDTA before staining with CD3 CD3 (557597, APC mouse anti-human, clone SP34-2, 14 µL), CD4 (560836, Alexa FluorR 700 mouse anti-human, clone L200, 5 µL), and CD8 (560179, APC-H7 mouse anti-human, clone SK1, 5 µL) antibodies from BD biosciences. After fixation and permeabilization, cells were stained with IFNγ (559327, PE mouse anti-human, clone B27, 20 µL) and, TNFα (557647, PE-CyTM7 mouse anti-human, clone Mab11, 5 µL) antibodies from BD Biosciences, CD137 (130-119-886, VioBright FITC mouse anti-human, clone 4B4-1, 2 µL), and CD154 (130-113-609, VioBlue mouse anti-human, clone 5C8, 2 µL) antibodies from Miltenyi Biotec.
forFor anti-LASV IgM, IgM µ-chain (SAB3700778, Sigma-Aldrich, 5 µg/mL) coated Maxisorp plates were incubated with lysates of LASV Josiah-infected Vero E6 cells before the addition of diluted plasma (1:100, 1:400, and 1:1,600). Plates were then treated with mouse anti-LASV monoclonal antibodies (a kind gift of P. Jahrling, USAMRIID) and peroxidase-conjugated anti-mouse antibodies (SAB3701029, Sigma-Aldrich, 1:20,000). For anti LASV-IgG, Polysorp plates were first coated with lysates of LASV Josiah-infected Vero E6 cells or recombinant LASV Josiah NP or GP (Zalgen) and then incubated with plasma dilutions (1:250, 1:1,000, 1:4,000, and 1:16,000). Plates were treated with peroxidase-conjugated antibodies against nonhuman primate IgG (SAB3700766, Sigma-Aldrich, 1:5,000). MeV-specific IgG antibody titers were also determined. as described elsewhere 23. Briefly, Maxisorp plates were coated with inactivated MeV antigens (PR-BA 102, Jena Biosciences) and then incubated with diluted plasma (1:250, 1:1,000, 1:4,000, and 1:16,000. Final staining was performed using peroxidase-conjugated antibodies against nonhuman primate IgG (SAB3700766, Sigma-Aldrich, 1:5,000).

Validation

All antibodies used in cytometry analyses were validated in prev/sous studies (Mateo et al., Science Translational Medicine, 2019 & 2021). They were selected based on their known cross-reactivity with cynomolgus macaques as mentioned by the company web sites. Anti-monkey IgG HRP was tested in house to verify the reactivity with cynomolgus antibodies and evaluate the best dilution. Anti-rabbit and anti-monkey alkaline phosphatase antibodies are in house validated for titration experiments. Anti-mouse HRP is provided by the manufacturer for western blot applications and validated in house to provide efficient signal. Anti-monkey IgG was provided as suitable for flow cytometry by the manufacturer and all controls in the experiment confirmed this.

Eukaryotic cell lines

Policy information about cell lines and Sex and Gender in Research

Cell line source(s)	VeroE6 cells ref CRL-1586 were obtained from ATCC.
Authentication	As provided by ATCC, no further authentification was performed.
Mycoplasma contamination	VeroE6 cells were used to titrate Lassa virus were tested negative for mycoplasma (Mycoalert, Lonza).
Commonly misidentified lines (See ICLAC register)	No commonly misidentified cell lines were used in this study.

Animals and other research organisms

Policy information about studies involving animals; ARRIVE guidelines recommended for reporting animal research, and Sex and Gender in Research

Laboratory animals	The animals were all 2.5- to 3-year-old male cynomolgus monkeys (Macaca fascicularis) from Mauritius Island weighing 3.5 to 4 kg at the time of vaccination.
Wild animals	The study does not involve wild animals.
Reporting on sex	All animals in this study were male. The sex only depends on availability and female could have been used instead. However, male and female cynomolgus monkeys are not mixed in an experiment to avoid any sexual behaviour that may harm the proper conduct of the experiment in the 14 facility.

Field-collected samples | The study does not involve samples collected from the field.

Ethics oversight | All procedures were approved by the Comité Régional d'Ethique en Matière d'Expérimentation Animale de Strasbourg (2018100414445313) and the Comité d'Ethique pour l'Expérimentation Animale CELYNE (2020061215142330).

Note that full information on the approval of the study protocol must also be provided in the manuscript.

Flow Cytometry

Plots

Confirm that:

☒ The axis labels state the marker and fluorochrome used (e.g. CD4-FITC).

☒ The axis scales are clearly visible. Include numbers along axes only for bottom left plot of group (a 'group' is an analysis of identical markers).

☒ All plots are contour plots with outliers or pseudocolor plots.

☒ A numerical value for number of cells or percentage (with statistics) is provided.

Methodology

Sample preparation

LASV-specific T cells were analyzed from 200 ul of fresh whole blood. PBS-EDTA (2 mM final concentration) supplemented with Live-Dead reagent (Molecular Probes) was added to samples before staining for CD3, CD4, and CD8 (BD Biosciences). Red blood cells were then lysed using PharmLyse (BD Biosciences). Cells were fixed and permeabilized using the FoxP3 staining buffer set (Miltenyi) before intracellular staining with antibody to IFNg, CD154, CD137 and TNFa (BD Biosciences or Miltenyi).
For surface staining only, surface antibodies were added to 50 uL of fresh whole blood and after staining, red blood cells were lyzed ans samples were fixed using Immunoprep (Beckman Coulter).
For surface and intracellular staining, surface antibodies were added to 50 uL of fresh whole blood and after staining, red blood cells were lyzed in BD Pharmlyse (BD Biosciences). Cells were then fixed and permeabilized using the FoxP3 staining buffer set (Miltenyi) before intracellular staining.

Instrument | Gallios cytometer (Beckman Coulter)

Software | Kaluza v2.1

Cell population abundance | Cells from whole blood were not sorted.

Gating strategy

For ICS studies, singlets were gated using FSC int/FSC tof, then dead cells were excluded with Live/Dead staining. Lymphocytes were gated using FSC/SSC parameters, CD3+ cells were gated to identify CD4+ and CD8+ T cells. These populations were then analyzed for IFNg, CD154, and CD137 staining.
For phenotyping of T cells, singlets were gated using FSC int/FSC tof, then CD45+ cells were selected using CD45/SSC parameters. Lymphocytes were then gated using FSC/SSC parameters, CD3+ cells were gated to identify CD4+ and CD8+ T cells. These populations were then analyzed for CD69, CD134, NKp80, CD279, CD28, CD27, CD45RA, Granzyme B, perforin, and KI-67 staining.

☒ Tick this box to confirm that a figure exemplifying the gating strategy is provided in the Supplementary Information.
